# The association between cervical cancer screening participation and the deprivation index of the location of the family doctor’s office

**DOI:** 10.1371/journal.pone.0232814

**Published:** 2020-05-15

**Authors:** Fanny Serman, Jonathan Favre, Valérie Deken, Lydia Guittet, Claire Collins, Michaël Rochoy, Nassir Messaadi, Alain Duhamel, Ludivine Launay, Christophe Berkhout, Thibaut Raginel

**Affiliations:** 1 Department of General Medicine, School of Medicine, Lille University, Lille, France; 2 Department of Public Health, University Hospital of Lille, Lille University, Lille, France; 3 Department of Epidemiological Research and Evaluation, University Hospital Caen, Caen, France; 4 INSERM U1086 « Anticipe », University Hospital Caen, Normandie University, Caen, France; 5 Department of General Medicine, Medical School, Normandie University, Caen, France; 6 Irish College of General Practitioners, Dublin, Ireland; The University of the South Pacific, FIJI

## Abstract

**Background:**

Cervical cancer screening rates are known to be strongly associated with socioeconomic status. Our objective was to assess whether the rate is also associated with an aggregated deprivation marker, defined by the location of family doctors’ offices.

**Methods:**

To access this association, we 1) collected data from the claim database of the French Health Insurance Fund about the registered family doctors and their enlisted female patients eligible for cervical screening; 2) carried out a telephone survey with all registered doctors to establish if they were carrying out Pap-smears in their practices; 3) geotracked all the doctors’ offices in the smallest existing blocks of socioeconomic homogenous populations (IRIS census units) that were assigned a census derived marker of deprivation, the European Deprivation Index (EDI), and a binary variable of urbanization; and 4) we used a multivariable linear mixed model with IRIS as a random effect.

**Results:**

Of 348 eligible doctors, 343 responded to the telephone survey (98.6%) and were included in the analysis, encompassing 88,152 female enlisted patients aged 25–65 years old. In the multivariable analysis (adjusted by the gender of the family doctor, the practice of Pap-smears by the doctor and the urbanization of the office location), the EDI of the doctor’s office was strongly associated with the cervical cancer screening participation rate of eligible patients (p<0.001).

**Conclusion:**

The EDI linked to the location of the family doctor’s office seems to be a robust marker to predict female patients’ participation in cervical cancer screening.

## Introduction

In 2012, cervical cancer was the second most common cancer in women worldwide after breast cancer [1] with 80% of cancers occurring in developing countries [2]. In France, cervical cancer is the twelfth most frequent cancer and was responsible for 1,102 deaths in 2012 (specific mortality rate of 1.8/100,000 women) [3], with the highest specific mortality in the Nord-Pas-de-Calais region [4], in which French Flanders is located. Cervical cancer results from the slow progression of dysplastic intraepithelial lesions of cervical cells following persistent infection by oncogenic human papillomavirus (HPV). Incidence of invasive cervical cancer can be dramatically reduced if precancerous lesions are treated. The French official guidance (citation: HAS) recommends cervical screening by Pap-smears every three years for women aged 25 to 65 [4,5] as this is widely associated with a reduction of both cervical cancer incidence and specific mortality [6–8].

Socioeconomic deprivation is a major risk factor for both lack of vaccination [9] and lack of regular gynecological follow-up [10] and as a consequence, lack of cervical screening [11,12]. According to a 2003 worldwide meta-analysis, being of disadvantaged socioeconomic status was associated with almost a 100% increased risk of cervical cancer development and with 60% increased risk of dysplasia [13]. Young women from a disadvantaged social background are less likely to be vaccinated, to have gynecological follow-up or to adhere to the screening program. In the USA in 2006, the cervical cancer mortality rate was doubled in the rather deprived state of Mississippi compared to the relatively affluent state of Rhode Island. [9]

In France, Pap-smears can be carried out by medical doctors (family doctors, gynecologists, medical biologists) and midwives. In 2010, 85% of Pap-smears were performed by gynecologists [5] although women most at risk of persistent oncogenic HPV infection were statistically more likely to be those who did not consult a gynecologist regularly. Only 53% of family doctors performed Pap-smears in 2013 [14]. Increasing the number of Pap-smears performed by family doctors (by sending free screening kits or by providing free training) could help in targeting unscreened women not being managed by a gynecologist [15]. Ample evidence shows that public health efforts must be targeted to family planning offices in deprived locations but, even though several studies correlate women’s socioeconomic status and cervical cancer screening [11,12,16–18], to our knowledge, only one French study, based on a telephone survey, analyzed the association between the socioeconomic status of the area of the doctor’s office and the cervical screening participation rate of female patients [19].

The main objective of this study was to measure the association between the deprivation index of the area in which the family doctor’s office was situated and the cervical screening rate in a multivariable analysis taking into account the gender of the doctor, the practice of Pap-smears by the doctor and the location (urban or rural) of the doctor’s office.

Moreover this study was a necessary preliminary to the PaCUDAHL-Gé [20] randomized clinical trial, assessing a HPV self-sampling device provided by the family doctor to female patients not participating in the usual opportunistic cervical screening program.

## Methods

### Ethics approval, consent to participate and support

This study is ancillary to the PaCUDAHL-Gé trial, which was approved by the French Agency for the Safety of Health Products (ANSM, 2015-A01331-48, 10/06/2015), by the Ethics Committee North-West III of Caen (2015–23, 02/03/2016) and is registered on ClinicalTrials.gov as NCT02749110. Participating family’s doctors gave their oral consent to the use of their data registered in the health insurance claim database when they were called to declare whether they were performing Pap-smears or not. No individual patient data were used in this article.

A fully-anonymized version of our database is publicly available at https://osf.io/cjurm/.

PaCUDAHL-Gé trial was granted by the French Ministry of Health (PREPS: LIC-14-14-0615, 12/19/2014) and sponsored by the University Hospital of Lille. For this study, only analysis and publication costs were covered by the grant.

### Design

The data for this study was collected from the claim database of the Primary Health Insurance Fund of French Flanders (between January 1^st^, 2013 and December 31^th^, 2014), from an individual telephone survey carried out in 2015 and from the 2015 deprivation index (based on the population census database of 2007).

### Population

**Fig 1:**
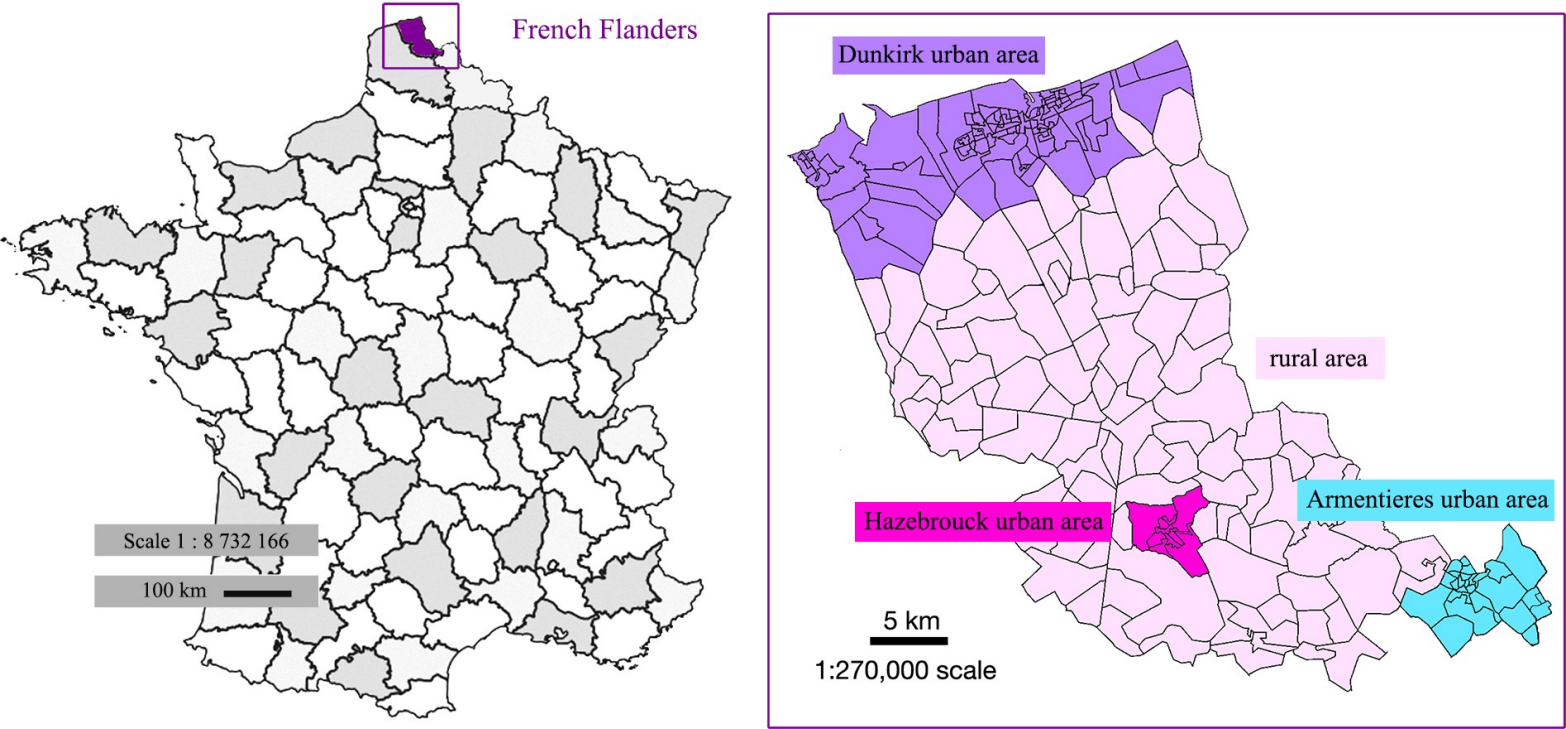
French Flanders divided in IRIS census units (right), in context of France (left), with urban and rural zones.

The population was the 402 doctors registered by the Health Insurance Fund of French Flanders [21]. French Flanders is the northern-most part of the *Nord* Department of France (see Fig 1).

French patients have to be registered on a doctor’s list (this doctor partially acting as a gatekeeper) to be fully reimbursed by health insurances. Doctors who had less than 100 female patients registered were excluded in order to avoid scenarios other than primary care and the possibility of practice instability (50 doctors were excluded among which there were 7 angiologists, 1 nutritionist, 2 ultrasound practitioners, 1 emergency doctor, 2 acupuncturists, 9 homeopaths, 2 politicians, 1 medical adviser of health insurance, 1 cosmetic surgery practitioner and 24 family doctors in early retirement or first year of practice). Doctors retired before December 31^th^ 2014 were also excluded. Refusing to answer the telephone survey was also a reason for exclusion.

### Individual level variables

For each family doctor, we collected from the health insurance database: the name and gender of the doctor, the postal address of the doctor office, the number of registered patients on January 19, 2015, the number of enlisted female patients aged from 25 to 65 years (theoretically eligible for cervical cancer screening) on January 19, 2015, and the number of these women who had at least one Pap test cytology refunded between January 1, 2013 and the December 31, 2014. The cervical cancer screening participation rate in 25-65-year-old women was computed for each doctor as the number of those who underwent at least one Pap-test during the monitored two years divided by the total number of women in the age bracket.

A telephone survey was carried out between January and July 2015. In order to maximize the response rate, each doctor was asked one single question: “Do you perform Pap-smears yourself in your practice?” A binary variable was allocated according to the yes/no answer received.

### Geographical area level variables

Each family doctor’s office was geotracked using National Geographic Institute (IGN) [22] mapping and attributed the code of the corresponding IRIS (regrouped statistical information blocks) as defined by the National Institute for Statistics and Economic Studies (INSEE) [23]. The IRIS is the smallest available statistical census unit in France allowing for studies on an intra-municipal level. IRIS divisions roughly match islets of 1,500 to 5,000 individuals with relatively homogeneous social characteristics. Villages or small towns constitute one single IRIS whereas larger towns are divided into several, resulting in around 50,000 IRIS in France. French Flanders is divided into 234 IRIS (see Fig 1).

Each IRIS census unit was associated with a binary variable of urbanization (1 if the census unit was part of the official grouping of conurbations of Dunkirk, Hazebrouck or Armentieres; 0 if not). This variable can account for accessibility in the first approximation since these conurbations are well-served by public transport.

Each IRIS census unit (and thus each family doctor’s office located in it) can be associated with its European Deprivation Index (EDI) [24] which is an ecological marker reflecting the individual deprivation experience in the area. This index was developed as the weighted sum of eleven variables quantifying fundamental needs associated with both objective and subjective poverty [24]:

Score EDI = 0.11 x “Overcrowding”
  +  0.34 x “No access to a system of central or electric heating”
  +  0.55 x “Non-owner”
  +  0.47 x “Unemployment”
  +  0.23 x “Foreign nationality”
  +  0.52 x “No access to a car”
  +  0.37 x “Unskilled worker-farm worker”
  +  0.45 x “Household with more than 6 persons”
  +  0.19 x “Low level of education”
  +  0.41 x “Single-parent household”:

These variables have been chosen by identifying all fundamental needs associated in a specific cultural context with both objective and subjective poverty, and then by retaining the ones that exist in several countries, and the ones that were the best fit for the deprivation indicator in a specific category (for example education or work). All variables can be calculated individually or for a census statistical unit. Finally, a multivariable logistic regression was run with all the chosen variables against individual deprivation indicators: regression coefficients of the model became weights in the above calculation of the deprivation index.

Variables exist in both European individual surveys of deprivation and national population censuses, allowing the EDI to be replicable in over 25 European countries and transposable in time.

We used EDI data, available in 2015, based on the 2007 French national census. The INSERM “Anticipe” unit based in Caen (Normandy, France) computed the EDIs where family doctors’ offices were located. Each IRIS census unit can be classified into quintiles according to its degree of deprivation (for entire mainland France: quintile 1[-5.3;-1.3], quintile 2[-1.3;0.7], quintile 3[-0.7;0], quintile 4:[0;0.9] quintile 5[0.9;20.5]) [24].

### Cartography

ImageJ [25] was used to create choropleth maps of quantitative variables on IRIS census units’ level in French Flanders: number of family doctors, EDI and cervical cancer screening participation rates.

### Statistical methods

Quantitative variables are expressed as mean ± standard deviation, median, IQR (interquartile range) and range; and the categorical variables are expressed as numbers (percentage). The association between each individual characteristic of family doctors and the screening participation rate was analysed using the Pearson correlation coefficient for continuous variables and a Student T-test for binary variables. The EDI is measured at the geographical level (IRIS). Therefore, the association between EDI and the screening participation rate was analysed using the mixed model with the geographical level as random effect. All the variables were introduced in a multivariable mixed linear regression with the screening participation rate as a dependent variable, and with doctors’ characteristics and EDI as independent variables. The geographical level (IRIS) was considered as random effect. A backward stepwise selection procedure at 0.2 level was performed to identify the subset of variables linked to the cervical cancer screening participation rate. The adjusted coefficients with the 95% confidence intervals were computed. All statistical tests were two-sided and performed at the 0.05 level. Data were analysed by the biostatistics department of the University Hospital of Lille, using SAS® software (version 9.3, SAS Institute Inc., Cary, NC, USA).

## Results

### Demography

**Fig 2:**
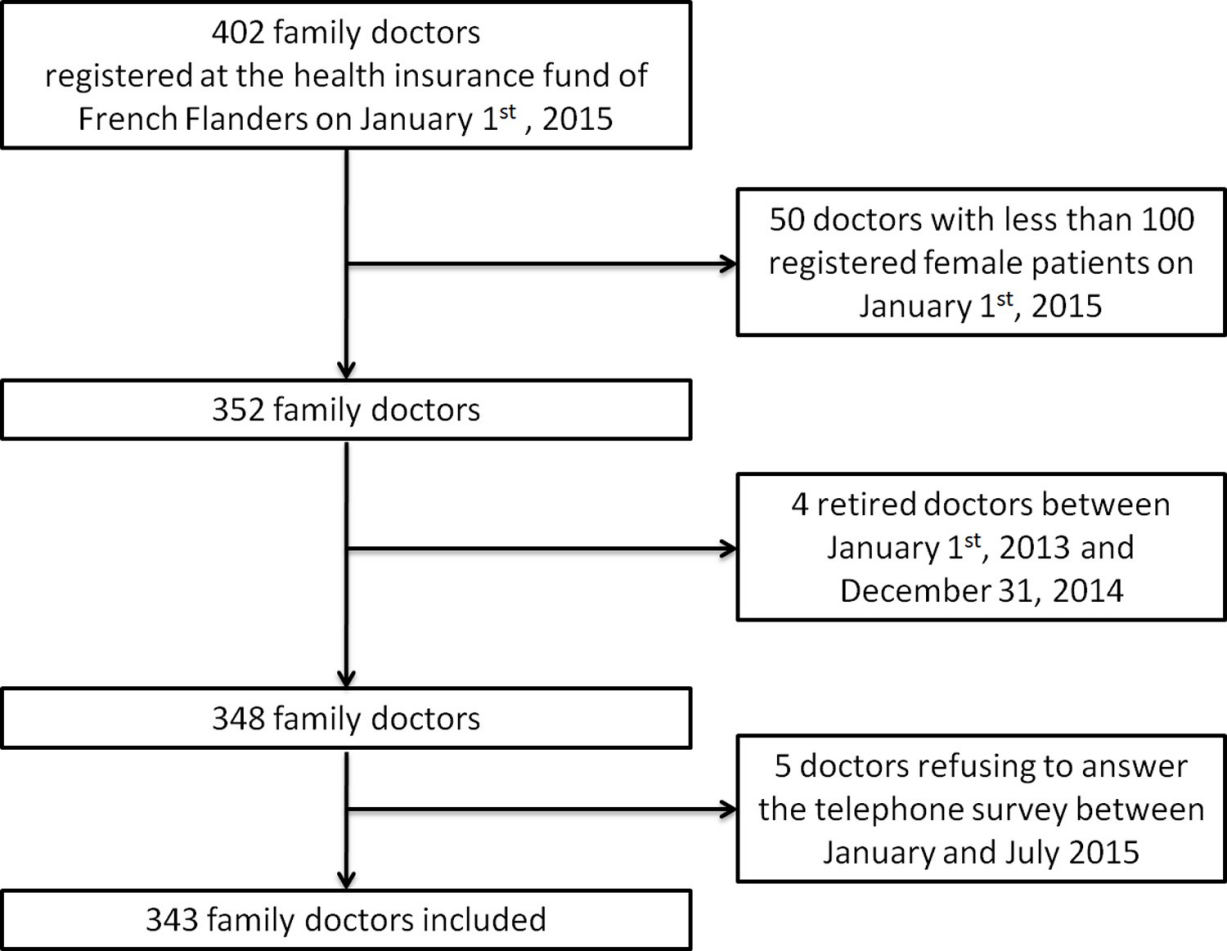
Flow chart of family doctors.

Among the 402 family doctors registered at the health insurance fund of Flanders, 50 were excluded because they had less than 100 female patients on their patients list and four were excluded due to retirement. Of the 348 included family doctors, five refused to answer the telephone survey (response rate: 98.6%). In total, 343 family doctors were included in the analysis, managing 128,175 listed female patients, 88,152 of whom were aged 25–65 years (see Fig 2 and Table 1).

**Table 1:** Descriptive results among family doctors.

Family doctors, n (%)	Characteristics	Total	Urban	Rural
		343 (100)	223 (100)	120 (100)
**gender of the doctor, n (%)**	Male	**269 (78.4)**	176 (78.9)	93 (77.5)
	Female	**74 (21.6)**	47 (21.1)	27 (22.5)
**practice of Pap-smears by the doctor, n (%)**	Yes	**182 (53.1)**	102 (45.7)	80 (66.7)
	No	**161 (46.9)**	121 (54.2)	40 (33.3)
**distribution of doctor’s office location, n (%)** (among the national quintiles of the European Deprivation Index*)	1st quintile (less deprived)	**21 (6.1)**	13 (5.8)	8 (6.7)
	2nd quintile	**21 (6.1)**	9 (4.0)	12 (10)
	3rd quintile	**67 (19.5)**	37 (16.6)	30 (25)
	4th quintile	**69 (11.4)**	34 (15.2)	35 (29.2)
	5th quintile (most deprived)	**165 (48.1)**	130 (58.3)	35 (29.2)
**doctor office’s location, n (%)**	rural area	**120 (35.0)**	-	120 (100)
	urban area	**223 (65.0)**	223 (100)	-
	Dunkirk	**148 (43.1)**	148 (66.4)	-
	Hazebrouck	**17 (5.0)**	17 (7.2)	-
	Armentieres	**58 (16.9)**	58 (26.0)	-
**doctors registered patients (all of them), N**	total	**239312**	160620	78692
	median (IQR)	**668 (492–852)**	684 (503–855)	616 (478–789)
	mean (± SD)	**698 (± 290)**	720 (± 302)	656 (± 260)
	values range	**[139–1845]**	[139–1845]	[202–1378]
**doctors registered women patients, n**	total	**128175**	86435	41740
	25–65 women patients	**88152**	59029	29123
	25–65 screened w. patients	**37255**	24424	12831
	median (IQR)	**352 (264–450)**	366 (278–463)	323 (242–422)
	mean (± SD)	**374 (± 157)**	388 (± 163)	348 (± 141)
	values range	**[100–1104] [100:1104]**	[100–1104]	[134–774]
**cervical cancer screening rate, %**	overall rate **	**42.3**	41.4	44.1
	median (IQR)	**44 (39–48)**	42 (38–47)	45 (42–49)
	mean (± SD)	**43 (± 7)**	43 (± 7)	45 (± 6)
	values range	**[20–62]**	[20–62]	[28–60]
**European Deprivation Index of office’s location**	median (IQR) mean (± SD)	**1.9 (-0.6–3.7) 2.2 (± 3.7)**	2.7 (-0.5–4.4)	0.9 (-0.9–2.6)
	values range	**[-4.3–15.6]**	[-3.8–15.6]	[-4.3–5.9]

Keys:

SD = standard deviation

IQR = interquartile interval

% = percent

n = number

* Quintiles of European Deprivation Index for entire mainland France are:

quintile 1[-5.3;-1.3], quintile 2[-1.3;0.7], quintile 3[-0.7;0], quintile 4:[0;0.9] quintile 5[0.9;20.5] [24]

** Overall cervical cancer screening rate is the total rate in the studied area namely 25–65 screened female patients divided by total of 25–65 female patients

**Fig 3:**
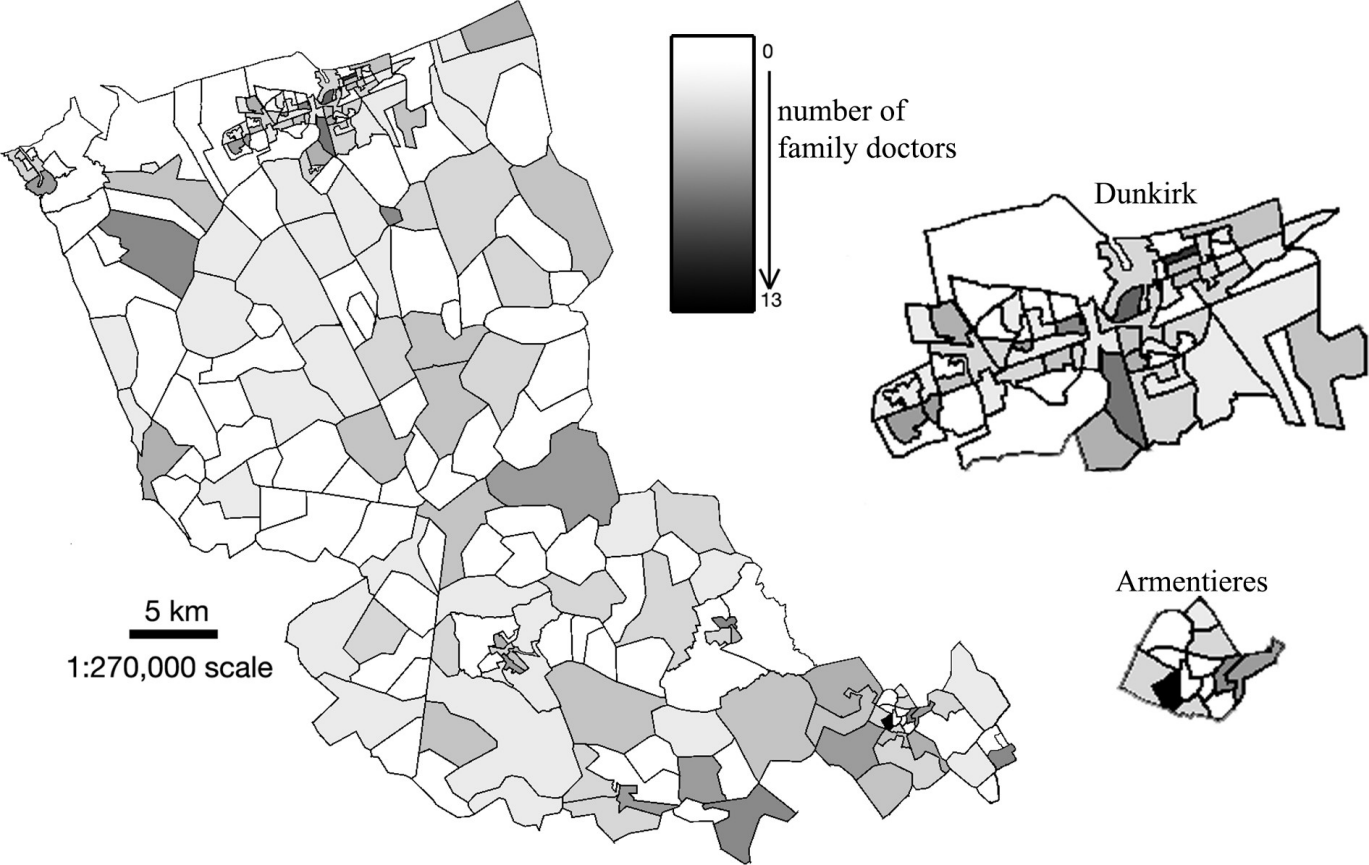
Family doctors’ density with blown-up images of Dunkirk and Armentieres.

The majority of family doctors were male (78.4%), had an office located in an urban area (65.0%) and about half (53.1%) performed Pap-smears themselves. Less than half of family doctors (48.1%) had their office in the most deprived areas (EDI within the fifth national quintile). More than half of the rural IRIS did not have any family doctor (Table 1 and Fig 3).

The overall cervical cancer screening participation rate (namely the total number of 25–65 screened female patients divided by the total number of 25–65 female patients) over two years was 42.3% (Table 1).

The European Deprivation Index median value in French Flanders was 1.9, meaning that the region is slightly more disadvantaged than the country as a whole (whose median is 0.0). More precisely rural parts of French Flanders are socioeconomically close to the whole country whereas urban areas are more disadvantaged.

**Fig 4:**
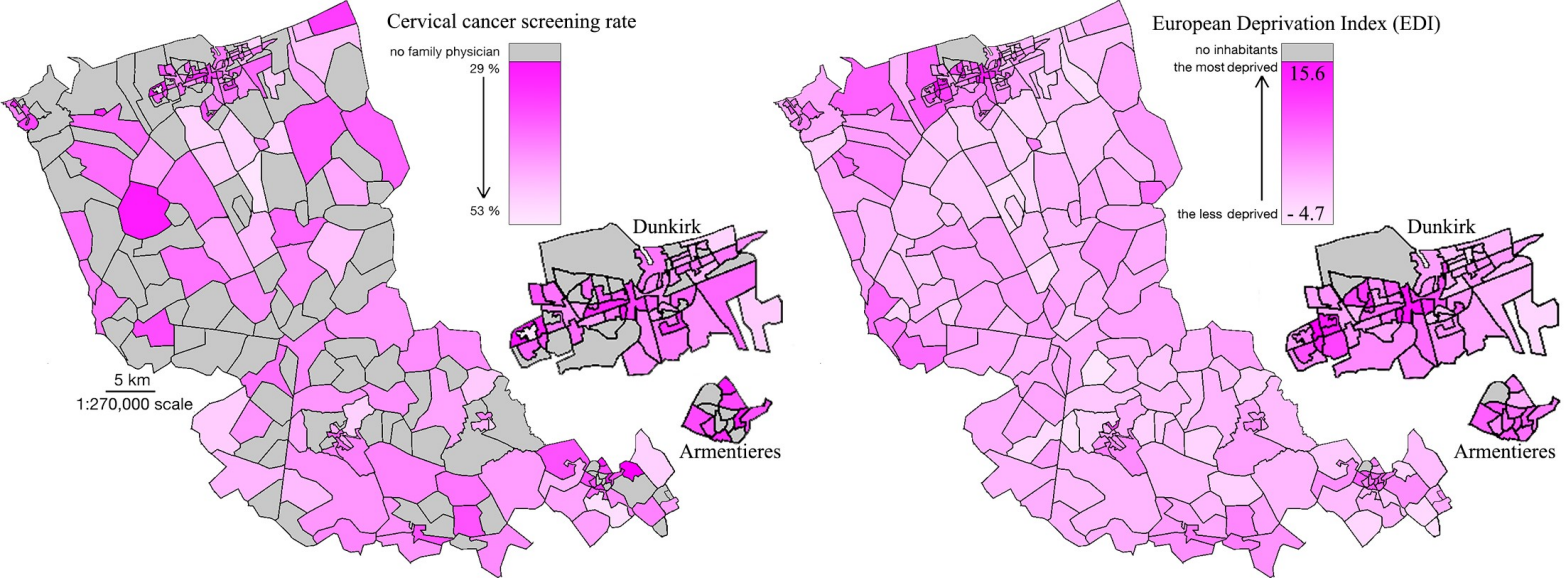
Cervical cancer screening participation rate (left); socioeconomic levels (right) in the French Flanders with blown-up images of Dunkirk and Armentieres.

Both the most deprived areas and the lowest cervical cancer screening rates were found in the urban zones where socioeconomic disparities were more evident (Table 1 and Fig 4).

### Main result

In univariate analysis (Table 2), the association between the socioeconomic status of the dwelling area of the family doctor office and the cervical cancer screening participation rate of female patients was significant (p<0.0001). Increasing one unit of the EDI (towards lower socioeconomic status on a 20-point scale) would lower the cervical screening participation rate by 0.6% (95% CI: 0.8 to 0.4).

**Table 2:** univariate analysis of the cervical cancer screening participation rate.

Variable	values	p-value*
number of registered patients (men, women)	-0.030^+^	0.58
number of registered women	0.016^+^	0.77
gender of the doctor: woman	45.19 ± 6.90 ^++^	**0.009**
man	42.82 ± 6.86 ^++^	
practice of Pap-smears by the doctor: yes	44.43 ± 6.66 ^++^	**0.002**
no	42.09 ± 7.03 ^++^	
doctor office location in urbanized location	-2.429 (-4.210 to -0.649) ^+++^	**0.008**
EDI	-0.596 (-0.805 to -0.388) ^+++^	**< .0001**

Keys:

EDI: European Deprivation Index

Values: + Correlation coefficient

++ Mean ± standard deviation

+++ Regression coefficient from linear mixed model with 95% confidence interval

*p-value: Unadjusted p-value for the association with the cervical cancer screening participation

The difference was also significant in univariate analysis when the family doctor was a female, when the family doctor was performing smears and when the doctor was in a rural area. Being registered with a women doctor, or a doctor who practices Pap-smears him/herself, or a doctor whose office is in a rural area would increase the average screening participation rate by respectively 2.4%, 2.3% or 2.4%. The differences in the cervical cancer screening rate depending on the number of enlisted patients in general (p = 0.58) or on the number of women registered patients in particular (p = 0.77) were not significant.

In multivariable analysis (Table 3), adjusting for gender of the doctor and practice of Pap-smears by the doctor did not alter the association between the cervical cancer screening participation rate and the EDI with the cervical screening rate still decreasing by 0.6% with every unit increase in EDI (towards lower socioeconomic status on a 20-point scale). Being registered with a doctor who practices Pap-smears him/herself would increase the cervical screening rate by 1.5%. However, being registered with a female doctor or with a doctor in a rural area was not significantly correlated with an increase of the cervical screening participation rate.

**Table 3:** Multivariable analysis of the cervical cancer screening participation rate.

	Full model		Selected model	
Variable	Coefficient (95% CI)^+^	p-value	Coefficient (95% CI)^+^	p-value
number of registered women	0.002 (-0.002 to 0.007)	0.353		
gender of the doctor (woman versus man)	1.535 (-0.187 to 3.257)	0.080	1.495 (-0.226 to 3.217)	0.088
practice of Pap-smears by the doctor (yes versus no)	1.566 (0.099 to 3.031)	**0.036**	1.711 (0.266 to 3.156)	**0.020**
doctor’s office location (Urban versus Rural)	-0.870 (-2.600 to 0.859)	0.321		
EDI	-0.550 (-0.764to -0.335)	**< .0001**	-0.562 (-0.771 to -0.354)	**< .0001**

Keys:

EDI: European Deprivation Index

^+^ Regression coefficient from linear mixed model with 95% confidence interval

## Discussion

### Key results

Participation in cervical cancer screening is known to be associated with socioeconomic factors, the most deprived populations being the least screened. This study demonstrated that the EDI of the location of the family doctor’s office was also strongly associated with the cervical cancer screening participation rate of female patients enlisted with this family doctor, in a multivariable model including size of the doctor’s office, gender of the doctor, practice of Pap-smears by the doctor and location (urban or rural) of the office. Multivariable analysis also confirmed that the practice of pap-smears by the practitioner himself or herself was significantly associated with greater participation rates in cervical cancer screening. On the other hand, it showed a lack of significant association with the gender of the doctor.

Urban areas were demographically characterized by both a lower cervical cancer screening rate and a more disadvantaged socioeconomic environment. However, even if a correlation existed between those two variables in univariate analysis, it was not significant when all variables were included.

### Limitations

Key results of this study are very robust, since they are based on almost all the practicing family doctors of a large territory, with an almost exhaustive telephone survey (98.6%) and thus a low risk of selection bias. Data collected from the claim database of the HIF of French Flanders are comprehensive and of good and homogenous quality [26]. The geotracking of family doctors’ offices in the appropriate IRIS census unit was meticulous, and the corresponding EDI was computed by the INSERM unit “Anticipe” in Caen.

However, this study had several limitations, namely the backward stepwise selection procedure, the duration of the data collection, the individual predictors of cervical cancer screening participation considered, and other ecological predictors. The backward selection is a data driven procedure which could fail to identify the best subset of variables associated to screening participation rate and introduce bias in the estimation of coefficients. Nevertheless, no significant variable in bivariate analysis was removed and we checked at each of the elimination steps the stability of the estimated coefficients. Data collection regarding cervical cancer screening participation encompassed two consecutive years, whilst the recommended screening interval between two smears in France is three years [4,5,27]. To this point, cervical cancer screening participation rates used in this study can only be used to highlight associations with other factors: they can’t be used to illustrate the absolute coverage rate of family doctors’ female patients. The reasons for this limitation are many: 1) as shown above, they encompass two years instead of three (under-evaluation); 2) they consider that all women aged 25–65 should be screened, whilst women with a history of cervical neoplasia, those who have never had intercourse or those who have had a hysterectomy should be excluded (under-evaluation of about 10% [19]); 3) they only took into account women who had a Pap smear cytology reimbursed by the health insurance, whilst smears performed in hospitals or in maternal and child welfare centers were not considered (we have estimated this under-evaluation in our population of 4.5%); and 4) the French Flanders area was chosen for the PaCUDAHL-Gé study (and thus for this preliminary study) due to a low rate of cervical cancer screening participation [28].

Finally, only 75% of 25–65 women are included in the Primary Health Insurance database (24% of the population is insured by other organisations and 1% has no health insurance), and it is unclear how these included women compare to women nationally. For all these reasons, the resulting rate can’t be extrapolated *beyond women in French Flanders covered by the Primary Health Insurance database*.

As other individual associated factors of cervical cancer screening participation rates, we considered the gender of the family doctor and the practice of Pap smears by the doctor, as found in the literature [29–31]. Other individual factors are also associated with cervical cancer screening rates, such as the age of woman and level of education [12,16,17,32,33]. However we avoided inserting the level of education since it was already included in the construction of the EDI [24]. We did not have individual data about the ages of the female patients.

The literature shows that another ecological predictor for cervical cancer screening rates is the delivery of health services by a gynecologist [19,34] and also that educated women are preferentially managed by a gynecologist [10,34]. For these reasons, it seems very unlikely that not having inserted the accessibility to a gynecologist in our equation could overestimate the association between EDI and the cervical cancer screening participation rate.

We did not directly acknowledge geographical accessibility of the family doctor’s office since it was considered subjective data (accessible on foot, on bike, in a car…) but assumed that urbanized locations were more accessible than rural ones.

One can argue that females can easily cross boundaries to go to another family doctor. Although it is strictly true, it did not really weaken our study, the goal of which was to show the correlation of cervical cancer screening and deprivation around family doctor’s offices, instead of the socioeconomic status of participants.

### Interpretation

The association between the socioeconomic level of patients and their health behavior was expected [35,36]. It points to social health disparities, with a higher morbidity and a shorter life expectancy in less educated and more socioeconomically deprived populations, whatever the average health level of a country [37–39]. Regarding cervical cancer, this mainly affects the most deprived populations, due to a lack of primary prevention (HPV vaccination) and secondary prevention (cervical cancer screening by Pap tests) [9,10].

The new knowledge brought to the scientific evidence base by this study is the link between the secondary prevention health behavior in women regarding cervical cancer screening and the location of their primary health care physician. This association was independent from the gender of the doctor and the practice of Pap-smears by the doctor himself or herself.

The lack of significant association with gender in the multivariable analysis confirmed recent French results from a large observational cross-sectional multicentre national study [40].

Accessibility (at first approximation urbanization of the area) was not associated with a better participation rate. Due to the socioeconomic environment of urbanized areas, it was even associated with a lower participation rate in the univariate model; and the association was not significant if all variables were included.

These findings suggest two main conclusions. First of all, the performance of the family doctor in increasing the participation of women in cervical cancer screening, is less related to the characteristics (gender and practice of Pap-smears) of the doctor, but related mainly to the characteristics of the population inhabiting the area around the family physician’s office [41]. Secondary, health policies should prioritize educational and innovative initiatives (for example vaginal or urinary self-sampling for a HPV-test based cervical cancer screening) in family doctors' offices located in deprived areas, even if these locations do not lack accessibility or gynecologists or family doctors [41,42].

### External validity

As discussed in the limitations section, the participation rate for cervical cancer screening in this study can’t be nationally generalized due to the two years duration of data collection and the low rate of screening in the North of France. Nonetheless, the association of the participation rate with the deprivation marker (computed by the EDI) of the location of primary care practices was strong and based on a robust level of evidence; all the limitations of this study likely reduced the strength of the association. The external validity of this study was thus high and could be generalized widely to countries with a well-developed primary health care service delivery.

## Conclusions

A low socioeconomic profile in the neighborhood of the family practice is strongly associated with a low cervical cancer screening rate for the female patients of the practice, even taking into account the practitioner’s gender and performance of Pap-smears in the practice. Having a family physician who undertakes Pap-smears him/herself is associated with a better rate of cervical cancer screening. Thus, it seems essential to ensure that practitioners working in disadvantaged areas are used to practicing pap smears. On the other hand, areas with low socio-economic conditions would appear to be the priority areas where specific public health actions to promote cervical screening should be implemented.

Finally, this study is ancillary to the PaCUDAHL-Gé trial which assesses the effect on screening participation rate of providing an HPV self-sampling device to female patients not participating in the usual opportunistic cervical screening program. Depending on the results of the ongoing PaCUDAHL-Gé trial, this study could show that providing self-sampling devices to family doctors working in disadvantaged areas may increase both the overall cervical screening participation for French Flanders women and reduce the disparities in cervical cancer burden.

## List of abbreviations

ANSM: French Agency for the Safety of Health Products
CI: Confidence Interval
EDI: European Deprivation Index
HPV: Human Papilloma Virus
INSEE: French National Institute for Statistics and Economic Studies
INSERM: French National Institute of Health and Medical Research
IRIS: Regrouped Statistical Information Islets (Ilots Regroupés pour l'Information Statistique)
